# Insulin Therapy and Risk of Prostate Cancer: a Systematic Review and Meta-Analysis of Observational Studies

**DOI:** 10.1371/journal.pone.0081594

**Published:** 2013-11-25

**Authors:** Yan-bo Chen, Qi Chen, Zhong Wang, Juan Zhou

**Affiliations:** Department of Urology and Andrology, Ninth People's Hospital, School of Medicine, Shanghai Jiaotong University, Shanghai, China; University of North Carolina at Chapel Hill, United States of America

## Abstract

**Background:**

Previous observational studies have shown that insulin therapy may modify the risk of prostate cancer (PCa). However, these studies yielded controversial results. Thus, we performed this meta-analysis to determine whether insulin use was associated with PCa risk in patients with diabetes mellitus (DM).

**Method:**

A literature search was carried out in PubMed, EMBASE, and Cochrane Library Central database between January 1966 and January 2013. Fixed-effect and random-effect models were used to estimate pooled relative risks (RR) and corresponding 95% confidence intervals (CIs). Subgroup analyses and sensitivity analyses were also performed.

**Result:**

A total of 11 (10 cohorts, and one case–control) studies published between 2007 and 2013 were included in the meta-analysis, representing data for 205,523 male subjects and 7,053 PCa cases. There were five studies investigating the influence of insulin and other glucose-lowering agents on the risk of PCa , and six studies investigating the influence of glargine and non-glargine insulin. Insulin use was not associated with PCa risk when compared with other glucose-lowering agents (RR=0.89, 95% CI, 0.72-1.09). Use of insulin glargine did not contribute to susceptibility to PCa as compared with use of non-glargine insulin (RR=1.26, 95% CI, 0.86-1.84). Sensitivity analysis confirmed the stability of present results, since no individual study affected the pooled result significantly.

**Conclusions:**

Our results suggest that, there may be no significant association between insulin use and risk of PCa as compared with other glucose-lowering agents in patients with DM, and there was no substantial evidence for increase risk of PCa among insulin glargine users as compared to non-glargine insulin users. Further studies are warranted to validate these conclusions.

## Introduction

Prostate cancer (PCa) is the sixth leading cause of cancer death in males worldwide[1] and is the second leading cause of cancer death among American men [2]. The cause of PCa is not well known, but multiple risk factors have been identified, including age, race, and family history of PCa. Many putative risk factors, including androgens, diet, physical activity, sexual factors, inflammation, and obesity, have been investigated, but their roles in PCa etiology remain unclear [2]. The global prevalence of diabetes mellitus (DM) is rapidly increasing as a result of population ageing, urbanization and associated lifestyle changes[3,4]. Type 1 DM accounts for 5%–10% of the total cases of DM and type 2 DM accounts for 90%–95%. The association between PCa and DM is complex and numerous epidemiological studies have tried to ascertain the relationship between the DM and PCa. Some studies strongly suggested a positive link between DM and PCa in terms of mortality[5], incidence[6], and more advanced cancer[7].

Whether insulin treatment increases risk of cancer is an important issue because almost all patients with DM will eventually require insulin treatment[8]. The association between insulin and cancer growth is linked at the biological level through hyperinsulinemia. Insulin is known to promote cellular growth and proliferation, and receptors for insulin are highly expressed on various types of cancer cells[9,10]. Although several observational studies have investigated the association between insulin-treated DM and risk of PCa; however, the results were inconsistent. Carstensen and colleagues found that insulin use was associated with reduced risk of PCa[11]; however, the significant decreased risk was not observed by other researchers[12-15]. As a result, whether insulin therapy is a risk factor for PCa remains unknown. Formulations of exogenous insulin used to manage diabetes vary in their affinity for the insulin receptor, IGF-1. Insulin glargine, a long-acting analogue, has a higher affinity for IGF-1. Several in vitro studies showed that the mitogenic potency of insulin glargine was higher than other insulin[16,17]. A lot of observational studies have also investigated the differences in PCa risk between insulin glargine therapy and non-glargine insulin therapy [18-23]. These studies yielded different or even controversial results. Hence, we performed a meta-analysis of observational studies to evaluate the effect of insulin therapy on the risk of PCa in patients with DM. 

## Methods

### Literature Search

This meta-analysis was conducted following guidance provided by the Cochrane Handbook[24] and was reported according to the Preferred Reporting Items for Systematic reviews and Meta-Analyses guidelines(PRISMA)[25]. A literature search was carried out using PUBMED, EMBASE, and Cochrane Library Central database between January 1966 and January 2013. There were no restriction of origin and languages. Search terms included: ‘‘insulin’’, and “diabetes” or ‘‘diabetes mellitus” or “DM” and ‘‘cancer(s)’’ or ‘‘neoplasm(s)’’ or ‘‘malignancy(ies)’’. Because a lot of studies investigated insulin use and risk of different types of cancer together(not only for prostate cancer), we didn’t limit “prostate” or “prostatic” to avoid missing important articles. The reference lists of each comparative study included in this meta-analysis and previous reviews[26,27] were manually examined to identify additional relevant studies.

### Study selection

Two reviewers(CQ and CY) independently selected eligible trials. Disagreement between the two reviewers was settled by discussing with the third reviewer(WZ). Studies were selected if they met our criteria for study design (randomized controlled trials, cohort study or case-control study), population (patients with DM), outcome (PCa incidence reported) and one or both of our comparisons of interest(1): insulin vs. other glucose-lowering agents; and (2) insulin glargine vs. all other types of insulin. Studies without PCa assessment were excluded. When there were multiple publications from the same population, only data from the most recent report were included in the meta-analysis and the others were excluded. Studies reporting different measures of RR like risk ratio, rate ratio, hazard ratio (HR), and odds ratio (OR) were included in the meta-analysis. In practice, these measures of effect yield a similar estimate of RR, since PCa is not common among patients with DM [28-30].

### Data extraction

The following data was collected by two reviewers(CQ and CY) independently using a purpose-designed form: name of first author, publishing time, country of the population studied, study design, study period, duration of follow-up, number of male subjects, number of PCa cases, type of DM, mean age of participants, cancer diagnosis methods, data ascertainment methods, the relative risk (RR) estimates and its 95 % confidence intervals (CIs), confounding factors for matching or adjustments. 

### Methodological quality assessment

Newcastle-Ottawa scale(NOS) was used to assess the methodologic quality of cohort and case–control studies. The NOS contains eight items that are categorized three categories: selection (four items, one star each), comparability (one item, up to two stars), and exposure/outcome (three items, one star each). A ‘‘star’’ presents a ‘‘high-quality’’ choice of individual study. Two reviewers(CQ and CY) assessed the methodological quality independently. Disagreement between the two reviewers was settled by discussing with the third reviewer(WZ).

### Data synthesis and analysis

Heterogeneity was assessed using the Cochran Q and I^2^ statistics. For the Q statistic, a P value<0.10 was considered statistically significant for heterogeneity; for the I^2^ statistic, heterogeneity was interpreted as absent (I^2^: 0%–25%), low (I^2^: 25.1%–50%), moderate (I^2^: 50.1%–75%), or high (I^2^: 75.1%–100%)[31]. The overall analysis including all eligible studies was performed first, and subgroup analyses were performed according to (i) study design (prospective cohort, retrospective cohort and case–control study), (ii) Study population(the continents which the studies conducted: America, Europe, and Asia), (iii)control for confounding factors ( n ≥ 6, n ≤ 5), and (iv) effect size (hazard ratio, relative risk, or odds ratio) to examine the impact of these factors on the association. When substantial heterogeneity was detected, the summary estimate based on the random-effect model (DerSimonian –Laird method)[32] was reported, which assumes that the studies included in the meta-analysis had varying effect sizes. Otherwise, the summary estimate based on the fixed-effect model (the inverse variance method)[33] was reported, which assumes that the studies included in the meta-analysis had the same effect size. To test the robustness of association and characterize possible sources of statistical heterogeneity, sensitivity analysis were carried out by excluding studies one-by-one and analyzing the homogeneity and effect size for all of rest studies. To better investigate the possible sources of between-study heterogeneity, a meta-regression analysis was performed[34]. A univariate model was established, and then variables with P values ≥0.1 were entered into a multivariable model. Publication bias was assessed using Begg and Mazumdar adjusted rank correlation test and the Egger regression asymmetry test[35,36]. Further, the trim and fill method which estimates the number and results of potential missing studies resulting from publication bias was applied[37]. All analyses were performed using Stata version 11.0 (StataCorp, College Station, TX).

## Results

### Search results and characteristics of studies included in the meta-analysis

**Figure 1:**
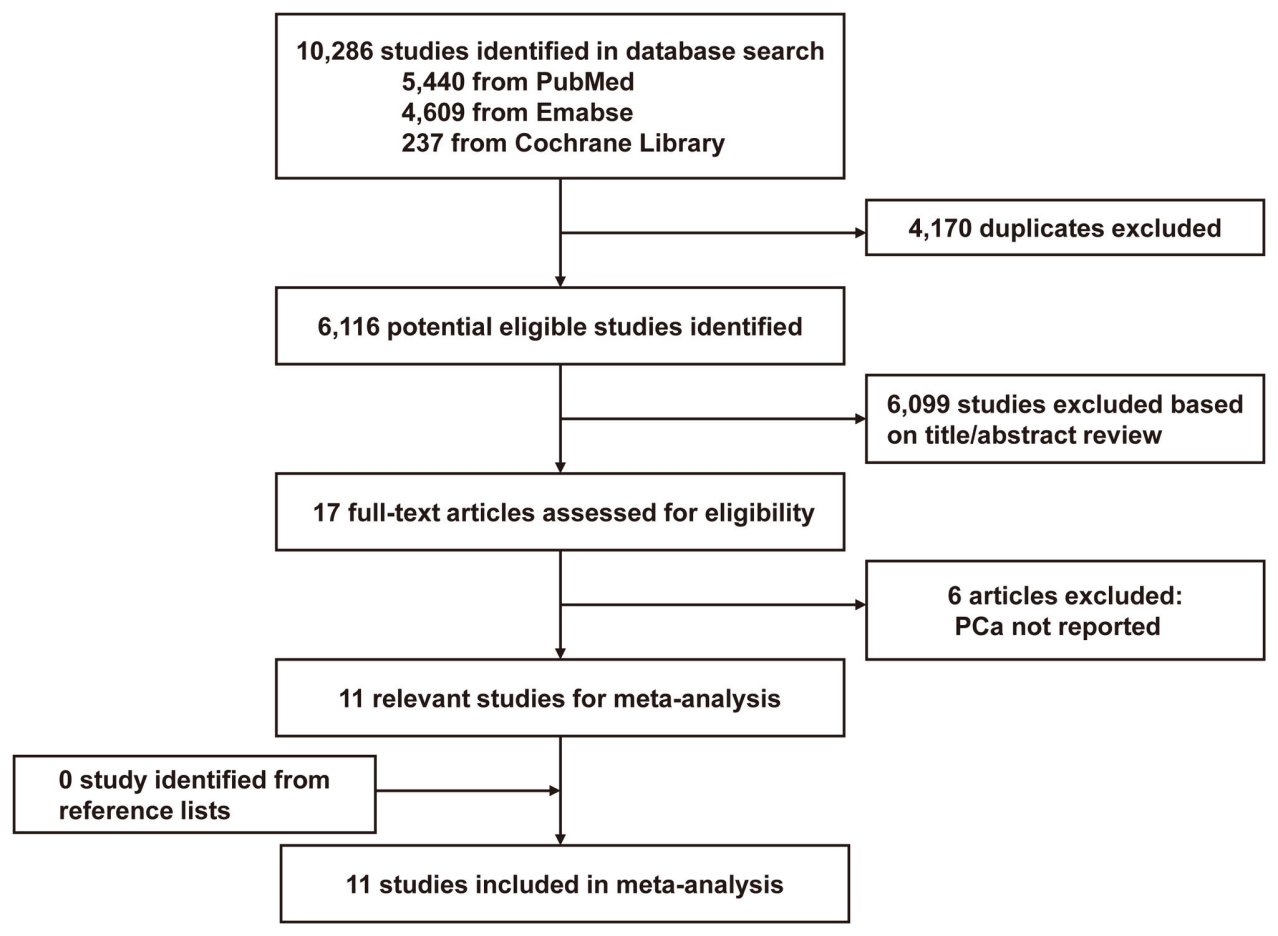
Flow diagram for study selection.

Figure **1** shows the flow diagram for study selection. A total of 10,286 citations were identified by the initial search. On the base of the titles and abstracts, we identified 17 full-text articles. After further evaluation, six studies were excluded for the reason of absence of data about PCa incidence. None study was identified from reference lists. At last, a total of 11 eligible studies published between 2007 and 2013 were identified, including 10 cohort studies [11-14,18-23], and one case–control study[15]. (Baseline data and other details of included studies are shown in Table 1). A total of 205,523 male subjects, including 7,053 PCa cases were involved. There were five studies investigating the effects of insulin and other glucose-lowering agents[11-15], and six studies investigating insulin glargine and non-glargine [18-23]. Of the 11 included studies, six studies were conducted in Europe[11,12,18,20,21,23], two in the USA[15,22], and the remaining three studies were conducted in Asia[13,14,19]. The NOS scores for the included studies ranged from 6 to 8, with a median 7; all these studies were deemed to be of a high quality (≥6) (shown in Table **1**). 

**Table 1:** Studies included in the meta-analysis.

Author	Year	Country	Study design	All male subjects	PCa cases	Mean Age(years)	Study period	Duration of follow-up (years)	Comparison	Starting time point of follow-up	Duration of insulin treatment(years)	Data Source	Type of diabetic	Confounders for adjustment	Adjusted risk estimate	NOS score
Gu Y	2013	China	Retrospective cohort	4,669	16	62.0	2001-2010	4.2(median)	Insulin vs no insulin use	from the diagnosis of DM	9.1 (mean)	Shanghai Diabetes Registry	Type 2	Age,sex,smoking status, diabetes duration, macrovascular,glycosylated hemoglobin,concomitant oral glucose lowering agents	RR: 2.07 (0.62–6.95)	7
Hsieh MC	2012	Taiwan	Retrospective cohort	31,568	587	61.4	2000-2008	NR	Insulin vs no insulin use	from the diagnosis of DM	NR	Taiwan’s National Health Research Institutes database	Type 2	Age, sex	OR: 0.89 (0.34–2.36)	6
Blin P	2012	France	Prospective cohort	866	20	68.8	2003-2010	NR	Insulin glargine vs human insulin	at the time of dispensing the first insulin prescription	NR	French national healthcare insurance system database	Type 2	Sex, type of diabetes, age,comorbidities, concomitant drug	HR: 0.41 (0.17, 0.99)	8
Carstensen B	2012	Denmark	Retrospective cohort	NR	2,582	60.5	1995-2009	5.3(mean)	Insulin vs no insulin use	from the diagnosis of DM	7-9	National diabetes register data	Type 1 and 2	Age,current date of follow-up, date of birth	RR: 0.79 (0.69–0.90)	6
Ruiter R	2012	Netherland	Prospective cohort	9,225	26	63.6	1998-2008	3.7(mean)	Insulin glargine vs non-glargine insulin	at the time of dispensing the first insulin prescription	1.9(median)	Dutch National Medical Register	Type 2	Age, sex, calendar time, hospitalization, unique drugs, other insulin use	HR: 2.74 (1.29–5.80)	8
Chang CH	2011	Taiwan	Retrospective cohort	25,594	38	61.8	2004-2007	1.9(mean)	Insulin glargine vs human insulin	at the time of dispensing the first insulin prescription	1.4(mean)	Taiwan National Health Insurance database	Type 2	Age, initiation year, sex, complication,concomitant drug, timing-varying medication use, dosage of insulin	HR: 2.59 (1.04–6.45)	7
Ljung R	2011	Sweden	Prospective cohort	53,674	663	65.0	2005-2008	2.5(mean)	Insulin glargine vs non-glargine insulin	from the diagnosis of DM	2.5(mean)	The Prescribed Drug Register, the Cancer Register, and the Causes of Death Register	Type 1 and 2	Age, sex	RR: 1.11 (0.81–1.52)	8
Morden NE	2011	USA	Retrospective cohort	25,660	2,072	77.4	2003-2008	1.9(mean)	Insulin glargine vs non-glargine insulin	from the diagnosis of DM	1.9(median)	Medicare Part D prescription drug program	Type 2	Age, race, diabetes complications, obesity, estrogen use, tobacco, income, comorbidities and insulin dose	HR: 1.14 (0.91 - 1. 43)	6
Colhoun HM	2009	Scotland	Prospective cohort	18,187	87	68.0	2002–2005	2.1(mean)	Insulin glargine vs non-glargine insulin	from the diagnosis of DM	2.1(mean)	Scottish Care Information-Diabetes Collaboration	Type 1 and 2	Prior cancer, type of diabetes, calendar year	HR 1.16 (0.16 -8.50)	7
Currie CJ	2009	UK	Retrospective cohort	32,261	301	62.0	2000–2009	2.4(median)	Insulin vs no insulin use	from the diagnosis of DM	6.2(mean)	The Health Information Network	Type 2	Age, sex, smoking status, diagnosis of a previous cancer	HR: 1.10 (0.79–1.52)	7
Koro C	2007	USA	Case-control study	3,819	643	NR	1997–2004	1.8(median)	Insulin vs no insulin use	from the diagnosis of DM	1.8(median)	Integrated Healthcare Information Services(IHCIS) managed care database	Type 2	Age,sex,calendar time, length of follow- up, years of recorded histroy in database before index date	OR: 0.77 (0.47–1.28)	6

PCa, Prostate cancer; NR, Not reported. NOS: Newcastle-Ottawa Scale

## Meta-Analysis Results

### Insulin vs. other glucose-lowering agents

**Figure 2:**
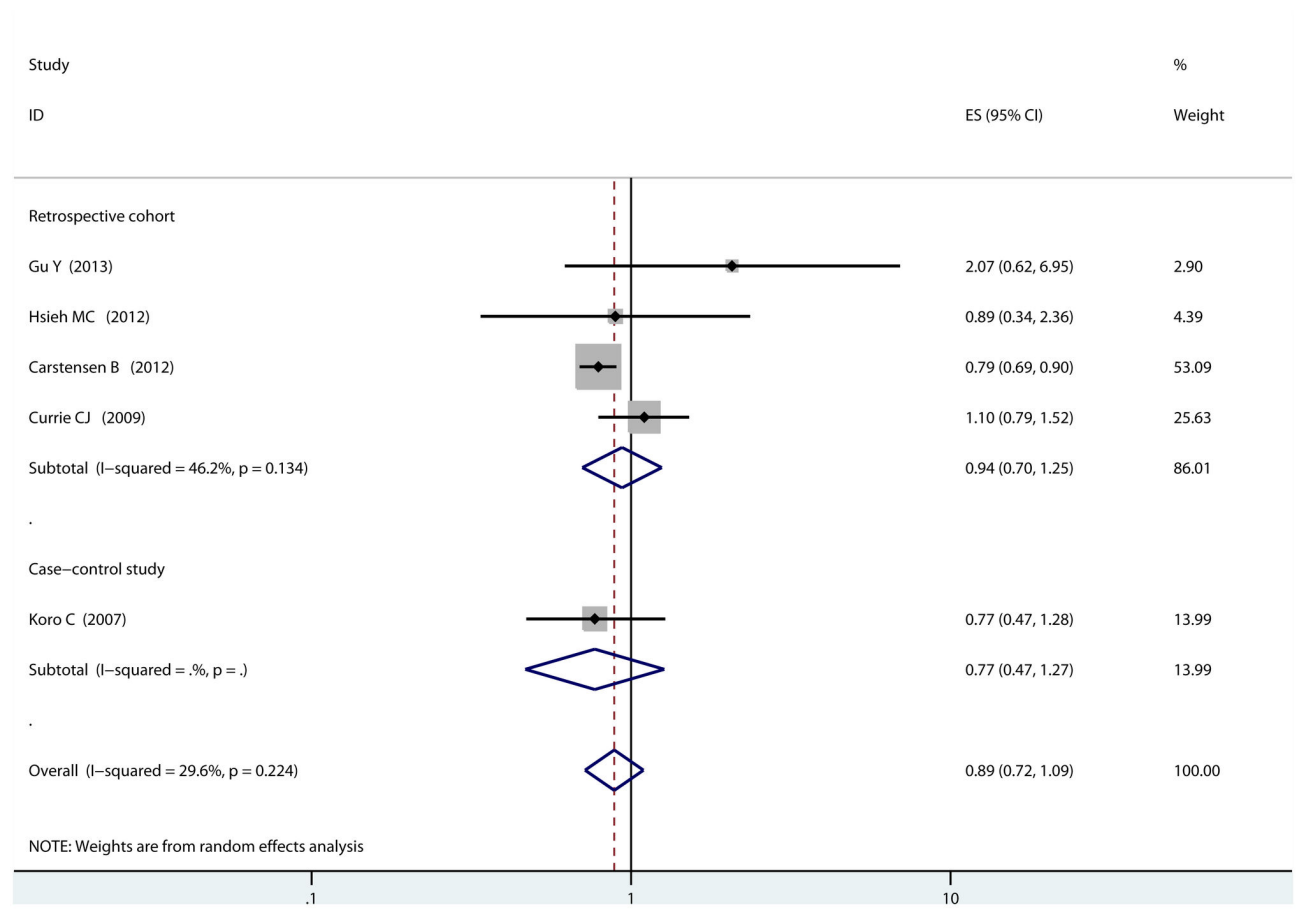
Forest plot: comparison of insulin vs. other glucose-lowering therapies and risk of prostate cancer.

Due to the presence of low heterogeneity (I^2^ =29.6%, p = 0.224), the fixed-effects model but not the random-effects model was chosen to provide a appropriate estimate of pooled RR and its 95% CI, and we found that, compared with other glucose-lowering agents, insulin use was not associated with risk of PCa (RR=0.89, 95% CI, 0.72-1.09, Figure 2). We found there was no statistically significant association between insulin use and risk of PCa among cohort studies (RR=0.94, 95% CI, 0.70-1.25) or case–control studies (RR=0.77, 95% CI, 0.47-1.27). When stratified studies by population, no statistically significant association was observed among studies conducted in Europe (RR= 0.90, 95% CI, 0.66-1.24), Asian (RR= 1.26, 95% CI, 0.56-2.82) or America (RR= 0.77, 95% CI, 0.47-1.27). We next examined whether adjustment of potential confounders could affect the pooled RR, no statistically significant association was observed among studies with higher control for potential confounders (RR=1.08, 95% CI, 0.43-2.70), as well as studies with lower control for potential confounders (RR=0.88, 95% CI, 0.69-1.13). When stratified the various studies by effect size, no statistically significant association was observed among studies which used HR (RR= 1.10, 95% CI, 0.79-1.53), RR (RR= 1.05, 95% CI, 0.44-2.49), and OR (RR= 0.79, 95% CI, 0.51-1.24) (Table 2).

**Table 2:** Insulin vs. other glucose-lowering agents and risk of prostate cancer.

	No. of studies	Pooled estimate		Tests of heterogeneity	
		RR	95% CI	P value	I^2^(%)
All studies	5	0.89	0.72-1.09	0.224	29.6
Study design					
Cohort study	4	0.94	0.70-1.25	0.134	46.2
Case-control study	1	0.77	0.47-1.27	―	―
Study population					
Europe	2	0.90	0.66-1.24	0.066	70.4
America	1	0.77	0.47-1.27	―	―
Asian	2	1.26	0.56-2.82	0.288	11.5
Adjusted for confounders					
n ≥ 6 confounders	2	1.08	0.43-2.70	0.138	54.5
n ≤ 5 confounders	3	0.88	0.69-1.13	0.183	41.1
Risk expression					
Relative risk	2	1.05	0.44-2.49	0.120	58.5
Hazard ratio	1	1.10	0.79-1.53	―	―
Odds ratio	2	0.79	0.51-1.24	0.790	0.0

To test the robustness of association and clarify possible sources of statistical heterogeneity, sensitivity analyses were carried out by excluding studies one-by-one and analyzing the homogeneity and effect size for all of rest studies. When we excluded the study by Carstensen B et al[11], the heterogeneity disappeared (I^2^ =0%, q = 0.423), which indicated that this study was the main source of heterogeneity. After excluding this study, the result was still insignificant (RR=1.02, 95% CI, 0.78-1.31). Moreover, no significant variation in combined RR was found by excluding any of other studies.

### Insulin glargine vs. non-glargine and risk of prostate cancer

**Figure 3:**
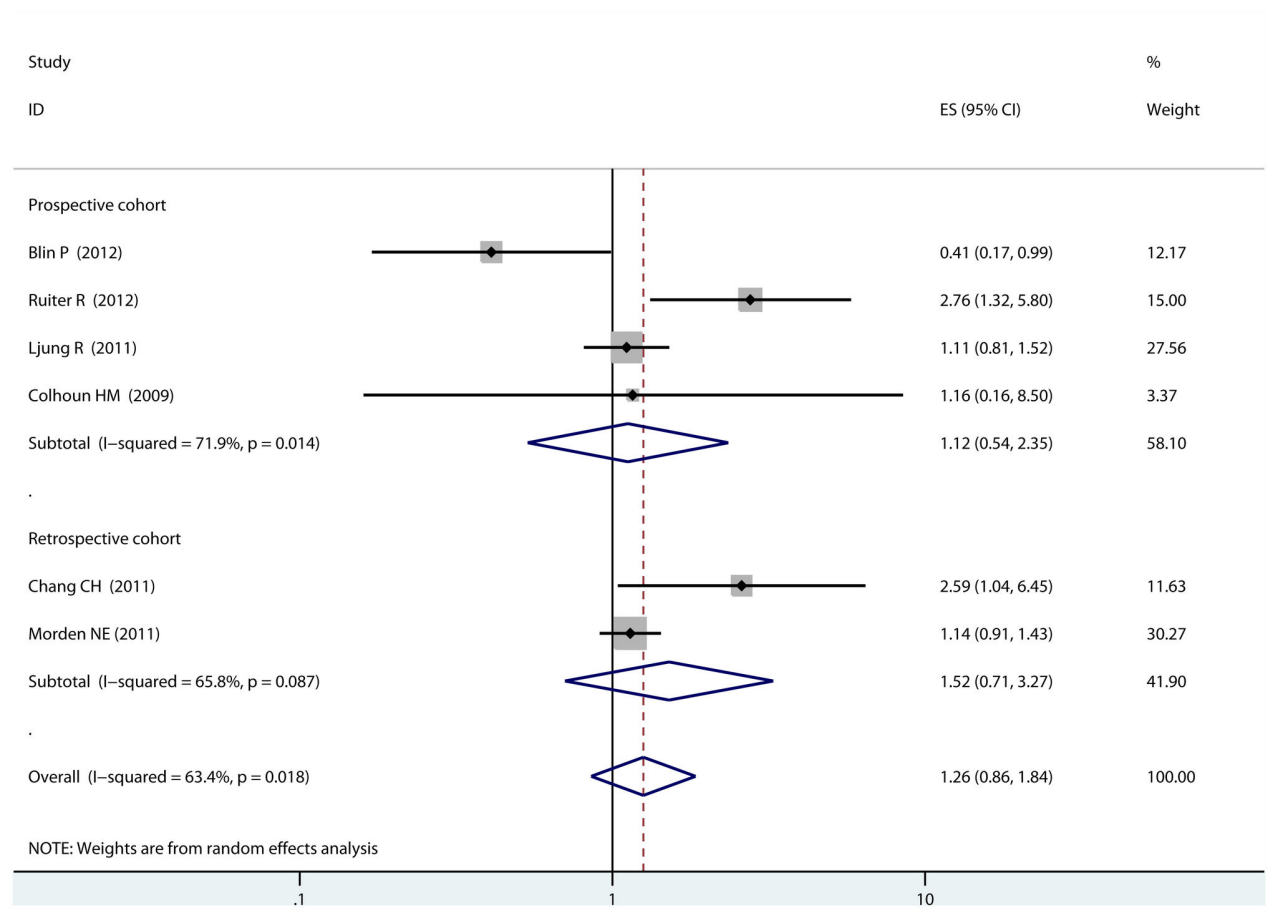
Forest plot: comparison of insulin glargine vs. non-glargine insulins and risk of prostate cancer.

Because of significant heterogeneity (I^2^=63.4%, q=0.018) was observed, the random-effects model was applied and we found that use of insulin glargine was not associated with increased risk of PCa in patients with DM as compared with use of non-glargine insulin (RR=1.26, 95% CI, 0.86-1.84, Figure 3). Study design did not affect the pooled result: prospective cohort studies (RR=1.12, 95% CI, 0.54-2.35), retrospective cohort studies (RR=1.52, 95% CI, 0.71-3.27) as compared with use of non-glargine insulin. When stratified eligible studies by population, no statistically significant association was noted among studies conducted in Europe (RR= 1.12, 95% CI, 0.54-2.35) or America (RR= 1.14, 95% CI, 0.91-1.43). However, compared to non-glargine insulin, use of insulin glargine was associated with a statistically increased risk of PCa in Asian population (RR= 2.59, 95% CI, 1.04-6.45). When we examined if adjustment of potential confounders could affect the combined RR, we observed that studies with higher control for potential confounders ( n ≥ 6) and studies with lower control (n ≤ 5) presented no statistically significant association (RR=1.83, 95% CI, 0.93-3.63 and RR=0.80, 95% CI, 0.39-1.68, respectively; Table 3). When stratified the various studies by effect size, no statistically significant association was observed among studies which used HR (RR= 1.33, 95% CI, 0.71-2.48), RR (RR= 1.11, 95% CI, 0.81-1.52). Sensitivity analysis confirmed the stability of present results, since no individual study affected the pooled result. 

**Table 3:** Insulin glargine vs. non-glargine and risk of prostate cancer.

	No. of studies	Pooled estimate		Tests of heterogeneity	
		RR	95% CI	P value	I^2^(%)
All studies	6	1.26	0.86-1.84	0.018	63.4
Study design					
Prospective cohort	4	1.12	0.54-2.35	0.014	71.9
Retrospective cohort	2	1.52	0.71-3.27	0.087	65.8
Study population					
Europe	4	1.12	0.54-2.35	0.014	71.9
America	1	1.14	0.91-1.43	―	―
Asian	1	2.59	1.04-6.45	―	―
Adjusted for confounders					
n ≥ 6 confounders	3	1.83	0.93-3.63	0.024	73.1
n ≤ 5 confounders	3	0.80	0.39-1.68	0.112	54.3
Risk expression					
Relative risk	1	1.11	0.81-1.52	―	―
Hazard ratio	5	1.33	0.71-2.48	0.009	70.4

### Meta-regression analysis

To better investigate the possible sources of between-study heterogeneity for the comparison of insulin glargine and non-glargine, meta-regression analysis was performed. Age, geographic area, publication year, control for confounding factors, follow-up time, study design, which may be potential sources of heterogeneity, were tested. However, meta-regression revealed that none of the above factors was responsible for the between-study heterogeneity. Because among the studies investigating insulin vs. other glucose-lowering agents, the study by Carstensen B et al[11] was found to be the main source of heterogeneity, meta-regression analysis was not performed.

### Publication bias

**Figure 4:**
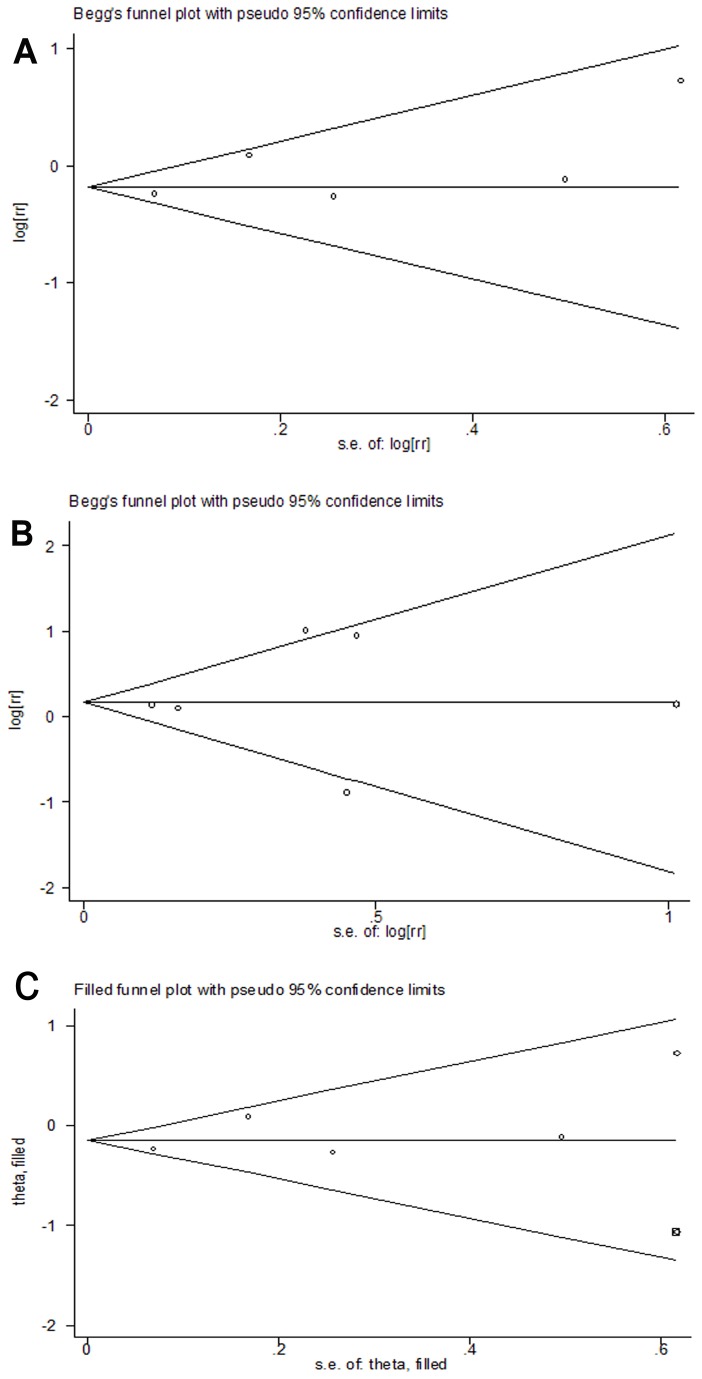
Funnel plots for publication bias. A: Funnel plot for studies investigating insulin vs. other glucose-lowering agents and risk of prostate cancer. No publication bias was observed ( P_Begg’s test_= 0.327, P_Egger’s test_ = 0.246) .B: Funnel plot for studies investigating insulin glargine vs. non-glargine and risk of prostate cancer. No publication bias was observed ( P_Begg’s test_= 0.851, P_Egger’s test_ = 0.718). C: Filled funnel plot of comparison of insulin vs. other glucose-lowering therapies and risk of prostate cancer. The filled diamonds represent one presumed missing study.

The potential publication bias was evaluated by funnel plot and Egger’s test. No visual publication bias was found in the funnel plot among studies investigating insulin and other glucose-lowering agents and risk of PCa (Figure 4A), or studies investigating insulin glargine vs. non-glargine and risk of PCa (Figure 4B). And Egger’s test suggested that no publication bias was detected among studies investigating insulin vs. other glucose-lowering agents (P=0.246), or studies investigating insulin glargine vs. non-glargine (P=0.718). Because the number of included study was too small, the trim-and-fill method was also implemented. For the comparison between insulin and other glucose-lowering agents, we showed that, if the publication bias was the only source of the funnel plot asymmetry, it needed one more study to balance the funnel plot(Figure 4C). However, the result didn’t changed significantly after one virtual study was appended under random-effects model (RR= 0.87, 95% CI, 0.69-1.10). For the comparison between insulin glargine and non-glargine insulin, trim-and-fill analysis did not indicate any missing study. 

## Discussion

Previous experimental studies have demonstrated that insulin can promote cellular growth and proliferation of prostate cancer cell and receptors for insulin are highly expressed on various types of cancer cell such as PCa[9,10]. Hyperinsulinism is also responsible for stimulating insulin-like growth factor-1 (IGF-1) secretion by the liver, which may act as a growth factor of multiple malign tumors *in vivo*, such as prostate carcinoma[38]. However, observational studies investigating insulin use and risk of PCa yielded different or even controversial results. In the present meta-analysis, we found that there was no statistically significant association between insulin use and risk of PCa as compared with other glucose-lowering agents in patients with DM. When we did subgroup analysis according to study type, no statistically significant association between insulin use and risk of PCa was found among cohort studies or case–control studies. When stratified studies by population, no statistically significant association was found among studies conducted in Europe, Asianor America. When we examined whether adjustment of potential confounders could affect the combined RR, it was observed that studies with higher control for potential confounders presented no statistically significant association, as well as studies with lower control for potential confounders. Sensitivity analysis revealed that the study by Carstensen B et al[11] was the main source of heterogeneity. The long follow-up duration (5.3 years) may be the reason why there was significant difference between the study by Carstensen B et al and other observational studies. The follow-up duration of all other studies was shorter than five years. Future studies should be adjusted for duration of DM. As we know, the duration of DM of patients treated with insulin may be longer than patients treated with other glucose-lowering agents. 

 Glargine (A21Gly, B31Arg, B32Arg human insulin), which differs from human insulin by replacing asparagine with glycine in position 21 of the A-chain and by carboxyterminal extension of B-chain by 2 arginine residues, is widely used as a long-acting insulin analogue in the treatment of DM. Previous experimental studies have shown that glargine may have potential carcinogenic effects for its higher mitogenic potency compared with non-glargine insulin[16,17]. However, others studies showed that the mitogenic potency of insulin glargine was similar to human insulin[39-41]. In the present meta-analysis, we found no substantial evidence for increase in PCa risk among insulin glargine users as compared to non-glargine insulin users. No statistically significant association between use of insulin glargine and risk of PCa was found among prospective cohort studies or retrospective cohort studies. When we examined if thorough adjustment of potential confounders could affect the combined RR, it was observed that both studies with higher control for potential confounders and studies with lower control presented no statistically significant association. When stratified the various studies by study population, no statistically significant association was noted among studies conducted in Europe and America. However, use of insulin glargine was associated with a statistically significant 159% increase in the risk of PCa as compared to non-glargine insulin among studies conducted in Asia. So, the oncogenic effect of insulin glargine was pronounced in the Asian population. We should notice that there is only one study investigating the association between insulin glargine use and Pca risk among Asians, the number is rather low to draw firm conclusion. This result should be confirmed by more studies and the mechanism also should be in investigated in the future. 

The strength of the present meta-analysis lies in inclusion of 11 studies and 205,523 male subjects. Most of the included studies(8 of 11) were published after 2010. This study included our report of two different exposure comparisons measures (insulin vs. no insulin and insulin glargine vs. other insulins). However, limitations of this meta-analysis should also be noted. First, we did not search for unpublished studies, so only published studies were included in our meta-analysis. Therefore, publication bias may have occurred although no publication bias was indicated from both visualization of the funnel plot and Egger’s test. Second, we allowed for exposure to any combination of other medications, including oral glucose-lowering agents, which may leads to over- or under-estimation of the true cancer risk[12,42,43]. Nonetheless, some included studies adjusted for use of oral glucose-lowering agents to minimize the confounding effects of these drugs. Thirdly, because no included study did distinguish between type 1 and 2 DM (3 studies investigated the association between PCa risk and insulin use among patients with type 1 and 2 DM together, and the other 7 studies only investigated the association between PCa risk and insulin use among patients with type 2 DM, seen in table 1), we haven't done sub-group analysis according to the type of DM. Further, over 90% of individuals with diabetes will have type 2 diabetes, so the majority of participants included in our meta-analysis were type 2 diabetes. Studies which distinguish between type 1 and 2 DM are needed in the future. Fourthly, the association between glargine insulin and risk of PCa in Asian population and American population was performed with only one study. For definition this isn't a meta-analysis, so more studies are need to further investigate the association in the future. Finally, the follow-up duration were different among the included studies, and the duration of most studies were less than three years. 

In conclusion, our results suggest that, there may be no significant association between insulin use and risk of PCa as compared with other glucose-lowering agents in patients with DM, and there was no substantial evidence for increase in PCa risk among insulin glargine users as compared to non-glargine insulin users. More studies, especially high quality cohort studies with larger sample size, well controlled confounding factors and longer duration of follow-up are needed to confirm these conclusions.

## Supporting Information

Table S1 **PRISMA checklist.**
(DOC)
